# Hantavirus stability and inactivation

**DOI:** 10.1371/journal.pntd.0013781

**Published:** 2026-07-02

**Authors:** Léna Vandenabeele, Abraham Ayanwale, Thomas Pietschmann, Benjamin Erik Nilsson-Payant

**Affiliations:** 1 Division of Virology and Immunology, Department of Microbiology, Tumor and Cell Biology, Karolinska Institutet, Stockholm, Sweden; 2 Institute for Experimental Virology, TWINCORE, Centre for Experimental and Clinical Infection Research, A Joint Venture between the Helmholtz Centre for Infection Research and the Hannover Medical School, Hannover, Germany; 3 Cluster of Excellence RESIST (EXC 2155), Hannover Medical School, Hannover, Germany; 4 German Center for Lung Research (DZL), Pneumonia and Acute Lung Injury (PALI), Hannover, Germany; 5 German Center for Infection Research (DZIF), Partner Site Hannover-Braunschweig, Germany, Hannover, Germany; USAMRIID: US Army Medical Research Institute of Infectious Diseases, UNITED STATES OF AMERICA

## Abstract

Hantaviruses are zoonotic viruses that can cause highly pathogenic disease, including hantavirus cardiopulmonary syndrome (HCPS) and haemorrhagic fever with renal syndrome (HFRS), in humans with case-fatality rates of up to 50%. However, our understanding of the basic viral life cycle and the underlying causes of viral pathogenesis remains sparse, in large part due to a lack of molecular biology tools for hantaviruses and the need to work in high-containment laboratory facilities with these viruses. The stability and inactivation of hantavirus particles has been examined in some limited previous studies, however, a comprehensive, detailed and robust investigation of the stability of multiple hantaviruses has not been performed yet. Here, we investigated the kinetics of infectious Tula virus (TULV) particle production in Vero E6 cells and subsequent stability in cell culture media. In addition, we evaluated the stability of infectious virus particles in response to different physical and environmental stresses, including heat, freezing, dehydration and UV exposure, answering key questions about the environmental transmission potential of hantaviruses. Interestingly, we observed a remarkable stability of TULV when stored at room temperature or colder, as well as after dehydration, which suggests that hantaviruses could remain infectious for a sustained period of time after being secreted by their host species. Subsequently, we determined the ability of commonly used virus inactivation methods, including RNA and protein extraction buffers, to inactivate TULV both in a cell-free and cell-associated context and found that TULV was efficiently inactivated by all these methods similar to other enveloped RNA viruses. Finally, we successfully validated the complete inactivation using these inactivation methods using the highly pathogenic HCPS-causing New World Andes virus (ANDV) and the HFRS-causing Old World Hantaan virus (HTNV). These results provide valuable information about safe and effective inactivation methods of viral samples and about the environmental risk potential of hantaviruses.

## Author summary

Hantaviruses are ubiquitous rodent viruses and contain some of the most lethal known zoonotic viruses, including Andes virus (ANDV) and Hantaan virus (HTNV), with no FDA- or EMA-approved antiviral treatment or prevention options available. However, studying the molecular biology and pathogenesis of these viruses is significantly impeded by the need for biosafety level (BSL) 3 (or higher) containment facilities for most hantaviruses and a general lack of molecular biology tools. Our study provides a comprehensive analysis of the stability of infectious hantavirus particles in response to different physical and chemical stresses. We demonstrate that a diverse range of hantaviruses are effectively and quickly inactivated by commonly used generic viral inactivation methods. However, we also provide evidence for an inherent stability under environmental conditions that could enable prolonged transmission potential of hantaviruses, even after secretion from their host species. These data are essential information to design safe inactivation methods of infectious hantavirus material and offer insights into the environmental risks of hantavirus infections with implications for laboratory safety and public health measures.

## Background

Orthohantaviruses (henceforth called hantaviruses) are a genus of segmented negative-sense RNA viruses within the *Hantaviridae* family that contain some of the most lethal known human pathogens. Ordinarily, hantaviruses circulate in their natural hosts – primarily rodents, but also shrews, moles, bats and other small mammals – where they are not thought to cause any disease [1,2]. However, some hantaviruses are known to be able to zoonotically infect humans and cause serious illnesses. Some Old World hantaviruses – including Hantaan virus (HTNV) and Seoul virus (SEOV) – can cause haemorrhagic fever with real syndrome (HFRS) in humans, which in severe cases is characterized by hypotension, vascular leakage, acute shock, acute kidney failure and case-fatality rates of up to 15% [3]. In Europe, Puumala virus (PUUV) can cause a mild version of HFRS called Nephropathia epidemica (NE), which is associated with fever, gastrointestinal symptoms and impaired renal function and case-fatality rates of less than 1% [4]. New World hantaviruses – including Andes virus (ANDV) and Sin Nombre virus (SNV) – are known to cause a severe respiratory disease called hantavirus cardiopulmonary syndrome (HCPS), which is characterized by initial flu-like symptoms which can rapidly progress to severe respiratory distress, pulmonary vascular leakage and respiratory failure with mortality rates of up to 40% [5].

Natural host reservoir species shed and transmit viral particles in their excreta, *e.g.,* their saliva, urine and faeces and zoonotic infection of humans primarily occurs through the inhalation of these excreta, *e.g.,* in the form of aerosolized dust particles [1]. Humans usually are dead-end hosts for hantaviruses, but human-to-human transmission, including nosocomial infections, has been observed for ANDV, causing several local epidemics in Argentina and Chile [6–8].

While it is impossible to accurately estimate the burden of human hantavirus infections due to their often-asymptomatic nature or very generic flu-like symptoms in mild cases, it has been estimated that globally 60,000–100,000 annual cases are recorded every year, with the majority of cases caused by HTNV and SEOV in China [1]. While hantavirus cases have reduced over the past decades in China, over 1.5 million cases of HFRS were recorded between 1950 and 2007, with a peak of 115,804 cases in 1986 [9,10]. In Europe, PUUV is the main cause of human infection accounting for usually less than 10,000 confirmed cases of human infections, with Finland, but also Sweden and Germany reporting the majority of cases [4]. In the Americas, human infection with New World hantaviruses occur much more sporadically and only a few thousand cases in total (the majority in Argentina and Chile) have been recorded since the discovery of new World hantaviruses in 1993 [1,11]. It is important to note however, that the actual case numbers for all hantaviruses are likely to be significantly higher.

While efforts are ongoing to identify and develop specific antiviral treatment options and vaccines – including clinical trials using DNA vaccines and broadly neutralizing monoclonal antibodies, there are currently no FDA- or EMA-approved antiviral treatments or vaccines available [12]. In consequence of this and taking their severe pathogenic potential into account, most hantaviruses need to be handled in high containment facilities. This significantly hampers the ease of basic and translational research, as the virus can only be handled in select laboratory facilities and requires stringent inactivation methods in order to be retrieved from the high-containment facilities. It is therefore important to gain a better understanding of how infectious material safely and effectively can be inactivated and how durable infectious particles remain under different environmental stresses. Previous studies have sporadically evaluated inactivation methods, including disinfectants, fixatives, UV irradiation and heat stability, using widely varying evaluation methods with varying degrees of robustness [13–18]. In this study, we primarily utilized Tula virus (TULV) – a close relative of PUUV – which is mostly considered non-pathogenic and has only been associated with human disease in extremely rare cases [19–22]. We evaluated its ability to retain its infectious capacity in response to different environmental and physical stresses, including exposure to heat, UV irradiation, long-term exposure at different temperatures, freezing and dehydration. Furthermore, we sought to determine whether different inactivation and lysis methods commonly used in lab protocols are able to effectively inactivate TULV both in a cellular and cell-free context. Here, we provide novel and important findings about the inactivation potential of different detergents and lysis buffers that are compatible with different downstream analysis methods, as well as about the environmental stability of TULV under different conditions. Finally, we validated whether our key findings for TULV could be cross-applied to highly pathogenic HFRS-causing (HTNV) and HCPS-causing (ANDV) viruses.

## Methods

### Cell and virus culture

African green monkey kidney epithelial cells (Vero E6; ATCC, CRL-1586) were commercially obtained. Cells were maintained in Dulbecco’s Modified Eagle Medium (DMEM) supplemented with 10% fetal bovine serum (FBS), L-Glutamine, MEM Non-Essential Amino Acids and 100 U/ml penicillin and 100 μg/ml streptomycin and cultured at 37°C and 5% CO_2_.

Tula virus (TULV; *Orthohantavirus tulaense*) strain Moravia/Ma5302V/94 was a kind gift by Rainer Ulrich (Friedrich-Loeffler-Institut, Germany). Tula/ Moravia/Ma5302V/94 virus was originally isolated from the lungs of infected *Microtus arvalis* trapped in Tvrdonice, South Moravia (Czech Republic) by passaging once in a hantavirus-free laboratory *Microtus arvalis* animal and subsequently in Vero E6 cells [23,24]. Andes virus (ANDV; *Orthohantavirus andasense*) strain Chile-9717869 was a kind gift by Piet Maes (Université Libre de Bruxelles, Belgium). Andes/Chile-9717869 virus was originally isolated from the kidneys and lungs of an infected wild *Oligoryzomys longicaudatus* trapped during an outbreak of HCPS in Chile in 1997 by passaging in Vero E6 cells [25,26]. Hantaan virus (HTNV; *Orthohantavirus hantanense*) strain 76–118 was acquired from the European Virus Archive Global (EVAg) repository (Ref-SKU: 008V-EVA1471) through its partner institution BMC-SAS (Bratislava, Slovakia). Hantaan/76–118 virus was originally isolated from the lungs of an infected wild *Apodemus agrarius* in Songnaeri, North Korea, in 1976 and subsequently passaged in A549 cells [27,28].

All viruses were propagated in Vero E6 cells in DMEM supplemented with 2% FBS, L-Glutamine, MEM Non-Essential Amino Acids, 100 U/ml penicillin and 100 μg/ml streptomycin. Virus-containing cell culture supernatants were repeatedly collected between 5–14 days post-infection (dpi) and replaced with fresh infection media. Pooled viral stocks were purified and concentrated using Amicon Ultra-15 centrifugal filter units (100-kDa molecular weight cut-off). Infectious virus titres were determined by immuno-plaque assays as described below.

All work involving infectious ANDV and HTNV was performed in the biosafety level 3 (BSL-3) facility of the Hannover Medical School (MHH) in accordance with its institutional biosafety requirements.

### Immuno-plaque assay

Infectious viral titres were determined by immuno-plaque assays as previously described in Vero E6 cells [29]. Vero E6 cells were infected with sequential 10-fold dilutions of virus and overlayed with 1.2% Avicel CL-611 in MEM containing 2% FBS, 10 mM HEPES, MEM Non-Essential Amino Acids and 100 U/ml penicillin and 100 μg/ml streptomycin. At 7 days post-infection, cells were fixed for 30 min at room temperature in 3.7% formaldehyde and permeabilized for 30 min at room temperature in 0.5% Triton X-100. Infected cells were immunostained for 2 h at room temperature with anti-NP monoclonal antibody clones TULV1 [30] for TULV and ANDV (a kind gift by Rainer Ulrich, Friedrich-Loeffler-Institut) or B5D9 for HTNV (Progen, cat# B5D9-C). Immuno-stained plaques were stained with either an IRDye 800CW-conjugated anti-mouse IgG (LI-COR, cat# 926-32210) or an HRP-conjugated anti-mouse IgG (Invitrogen, cat# 62–6520) secondary antibody. Immuno-stained plaque assays were visualised using an Odyssey CLx imaging system (LI-COR) and analysed with the Image Studio software (LI-COR) for fluorescently labelled plaques or using KPL TrueBlue Peroxidase Substrate for HRP-labelled plaques.

### Viral propagation assay

The continuous production of infectious TULV particles in Vero E6 cells was measured over time as following. Vero E6 cells were infected in triplicates with TULV at an MOI of 0.001 in infection medium (DMEM supplemented with 2% FBS, L-Glutamine, MEM Non-Essential Amino Acids, 100 U/ml penicillin and 100 μg/ml streptomycin). At 1 h post-infection (hpi), the viral inoculum was removed and replaced with fresh infection medium. At the indicated time points either 5% or 100% of cell culture supernatant was collected and replaced with fresh infection medium. Collected viral samples were frozen and stored at -80°C before all infectious titres were determined by immuno-plaque assays as described above.

### Physical inactivation of cell-free viral particles

To determine the effects of freezing viral solutions, freshly thawed aliquots of virus were flash-frozen on dry ice before being rethawed. For thermal inactivation of viral particles, approximately 1 × 10^6^ PFU of virus were placed in PCR tubes and exposed to the indicated temperatures for the indicated time in a preheated thermocycler. To determine thermal stability of viral particles, approximately 1 × 10^6^ PFU of virus were stored in the dark at 4°C, 21°C or 37°C. At the indicated time points, aliquots were frozen and stored at -80°C. To determine the effects of dehydration on viral particles, approximately 1 × 10^6^ PFU of virus were placed in a sterile cell culture plate and left to air dry in a biosafety cabinet for approximately 2 h. After complete dehydration, dried viral solutions were stored for the indicated periods of time in the dark at 4°C, 21°C or 37°C before being reconstituted in 300 µl DMEM supplemented with 2% FBS, L-Glutamine, MEM Non-Essential Amino Acids, 100 U/ml penicillin and 100 μg/ml streptomycin, incubated at room temperature for 5 min and mixed extensively by pipetting. As a control approximately 1 × 10^6^ PFU of virus were placed in a sterile cell culture plate but reconstituted immediately without allowing to air-dry. For ultraviolet (UV) irradiation of viral particles, approximately 1 × 10^6^ PFU of virus were placed in a sterile cell culture dish and exposed to indicated amounts of short wavelength UV-C irradiation (254 nm) in a CX-2000 UV Crosslinker (UVP). The cell culture dishes were placed approximately 10 cm below the UV-C light source. Infectious viral titres before and after all treatments were determined in parallel by immuno-plaque assay as described above.

### Chemical inactivation of cell-free viral particles

For chemical inactivation of cell-free viral particles, approximately 1 × 10^6^ PFU of virus were mixed for 10 min at room temperature with the chemicals at the final concentrations indicated in Table 1. PBS was used as a non-inactivating control. Commercial RNA extraction buffers were used at the indicated concentrations according to the manufacturers’ instructions. Subsequently, solutions were diluted in 10 ml PBS in order to reduce the concentration of cytotoxic chemicals and viral particles were concentrated using Amicon Ultra-15 Centrifugal Filter Units (100-kDa molecular weight cut-off). Retained concentrates (approximately 0.2 ml) were diluted once more in 10 ml PBS for an approximately 5,000-fold final dilution of inactivating chemicals and reconcentrated using the same Amicon Ultra-15 Centrifugal Filter Units. Retained solutions were titrated for infectious viral particles using immuno-plaque assays as described above.

**Table 1:** Inactivation methods for cell-free infectious particles.

*Chemical*	*Final concentration*	*Exposure time*
Ethanol	20%, 40%	10 min
Formaldehyde	1%	10 min
Sodium dodecyl sulfate (SDS)	1%	10 min
Nonidet P-40 substitute (NP-40)	0.5%	10 min
Triton X-100	0.1%, 1%	10 min
TRI Reagent	3:1 (75%)	10 min
Viral RNA Buffer (Zymo Research, cat# R1034-1)	2:1 (66.67%)	10 min
Buffer AVL (QIAGEN, cat# 19073)	4:1 (80%)	10 min
Binding Buffer (Roche, cat# 11858882001)	2:1 (66.67%)	10 min

### Chemical inactivation of intracellular viral material

For chemical inactivation of intracellular viral particles, at least 240,000 Vero E6 cells were infected at an MOI of 0.001 with TULV, HTNV or ANDV in DMEM supplemented with 2% FBS, L-Glutamine, MEM Non-Essential Amino Acids, 100 U/ml penicillin and 100 μg/ml streptomycin. At 9–12 dpi, cell culture supernatants were removed off infected cell monolayers and cells were treated for 30 min at room temperature with 100 μl of the indicated chemicals (Table 2) or PBS as a non-inactivating control. When necessary, samples were flash frozen at this stage. Subsequently, solutions were diluted in 50 ml PBS in order to reduce the concentration of cytotoxic chemicals and cells/lysates were concentrated using Amicon Ultra-15 Centrifugal Filter Units (100-kDa molecular weight cut-off). Retained concentrates (approximately 0.2 ml) were diluted once more in 10 ml PBS for an approximately 25,000-fold final dilution of inactivating chemicals and reconcentrated using the same Amicon Ultra-15 Centrifugal Filter Units. Retained solutions were used to infect approximately 240,000 naïve Vero E6 cells in 2 ml DMEM supplemented with 2% FBS, L-Glutamine, MEM Non-Essential Amino Acids, 100 U/ml penicillin and 100 μg/ml streptomycin. After 7–9 dpi, the concentration of infectious viral particles in the cell culture supernatants were determined using immuno-plaque assays as described above.

**Table 2:** Inactivation methods for intracellular infectious material.

*Chemical*	*Final concentration*	*Exposure time*
Formaldehyde	1%	30 min
Sodium dodecyl sulfate (SDS)	1%	30 min
Nonidet P-40 substitute (NP-40)	0.5%	30 min
Triton X-100	1%	30 min
Urea	8 M	30 min
TRI Reagent	100%	30 min
RNA Lysis Buffer (Zymo Research, cat# R1060-1)	100%	30 min
Buffer RLT (QIAGEN, cat# 79216)	100%	30 min

## Results

### TULV virion production and stability in cell culture media

**Fig 1:**
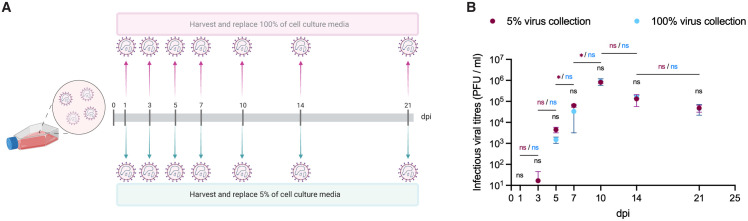
Infectious TULV particle production in Vero E6 cells. **(A)** Schematic of the experimental setup: Vero E6 cells were infected with TULV (MOI = 0.001) and either 100% or 5% of cell culture media were collected and replaced at the indicated time points before determining infectious viral titres. Conditions where 100% of the media is collected represent newly generated viral particles, whereas conditions where only 5% of the media is collected are an approximation for the total over time accumulated virus population. The image was created in BioRender, Guzyk, M. (2026) https://BioRender.com/zb9ri39. **(B)** Infectious viral titres of TULV at the indicated time points as determined by immuno-plaque assay. Individual dots represent independent biological replicates (n = 3). Statistical significance between time points was determined by two-way ANOVA followed by Šidák’s correction for multiple comparisons; statistical significance between collection methods was determined by two-way ANOVA followed by Turkey’s correction for multiple comparisons: * (p < 0.05), ns (p > 0.05).

Hantaviruses are relatively unique within RNA viruses in that they do not cause any significant viral-induced cytopathic effects (CPE). It has previously been shown that cells can be constitutively infected with hantaviruses and continuously produce and release viral particles into the cell culture media [30–32]. In order to get a better insight into the kinetics of infectious virion production and their subsequent stability in cell culture media during virus propagation, we infected Vero E6 cells with the mostly apathogenic TULV and measured the concentration of both nascently produced and cumulatively produced infectious viral particles in the cell culture supernatant over three weeks of infections (Fig 1A). We observed no infectious viral particles at 1 dpi at all and only in one replicate at 3 dpi. Low titres were detected after 5 dpi followed by a rapid increase in virion release and a clear peak in infectious viral particle production around 10 dpi. Viral titres subsequently decreased by 14 dpi and slowly kept decreasing until 21 dpi (Fig 1B). Notably, the cumulative and nascent infectious titres remained essentially identical to each other at each collection time. This indicates a short half-life of infectious viral particles in cell culture media at 37°C, meaning that the measured cumulative titres mostly reflect nascently produced viral particles and that older particles quickly lose their infectious potential.

### Stability and inactivation of TULV under physical stress

The ability of different viruses to survive different physical and environmental stresses and maintain infectivity differs vastly. Here, we tested the ability of TULV to withstand environmental stresses, including temperature, freezing, dehydration and UV exposure.

**Fig 2:**
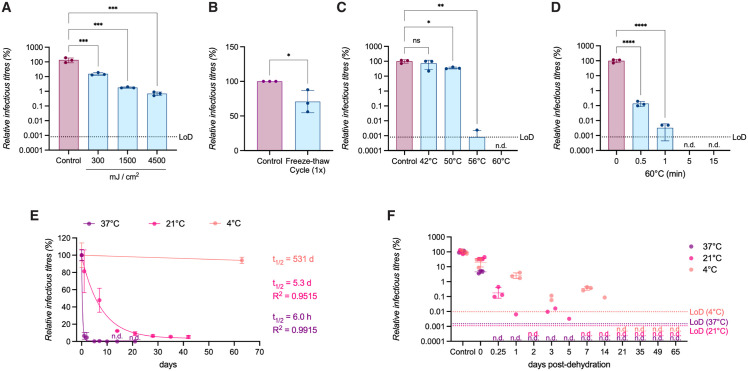
Environmental and physical stability of TULV particles. **(A)** Relative TULV infectious titres before and after UV (λ = 254 nm) irradiation at the indicated total doses. **(B)** Relative TULV titres before and after flash-freezing of virus stocks. **(C)** Relative TULV infectious titres before and after 15 min exposure to the indicated temperatures. **(D)** Relative TULV infectious titres before and after incubation at 60°C for the indicated times. **(E)** Relative TULV infectious titres after incubation of virus solutions at 37°C, 21°C or 4°C for the indicated times. Simple linear regression (for 4°C data) or nonlinear fit (for 37°C and 21°C data) was used to determine the half-life (t_1/2_) of infectious virus particles stored at 4°C (orange), 21°C (pink) or 37°C (purple). **(F)** Relative TULV infectious titres before dehydration (control) and after dehydration at room temperature and subsequent storage at 4°C, 21°C or 37°C. All graphs represent relative data from immuno-plaque assays normalised to the control condition, with dots depicting three independent biological replicates (n = 3) and error bars the standard deviation of the mean. The dotted lines represent the limit of detection (LoD) for the relevant graph. Data points below the LoD are not shown and indicated as not detected (n.d.) in the absence of any measurable data points. Statistical significance was determined by one-way ANOVA followed by Dunnett’s correction for multiple comparisons (for **A, C, D**) or by an unpaired t-test (for **B)**: **** (p < 0.0001), *** (p < 0.001), ** (p < 0.01), * (p < 0.05), ns (p > 0.05).

Ultraviolet (UV) irradiation is generally known to be able to inactivate RNA viruses, although the efficacy for different viruses varies depending on the wavelength and exposure time. Here we first exposed TULV in cell culture media to UV irradiation at 254 nm, resulting in a significant reduction (~10-fold) in infectious viral titres after a total dose of 300 mJ/cm^2^ UV exposure, with titres dropping even further at higher doses (Fig 2A). Next, we subjected TULV to a single freeze-thaw cycle and determined infectious viral titres before and after freezing. Infectious titres were only slightly reduced, suggesting that TULV might be relatively well equipped to tolerate freezing temperatures in the environment (Fig 2B).

Enveloped RNA viruses are generally known to be efficiently inactivated by heat denaturation. We therefore sought to determine how stable TULV remains at elevated temperatures and how quickly it is inactivated. After 15 min of exposure at 42°C, no significant loss of viral titre was detected. However, at 50°C approximately 35% and at 56°C less than 0.001% of infectious viral particles remained. At 60°C a complete loss of infectious viral titres under the limit of detection (>125,000-fold) was recorded (Fig 2C). In order to ascertain how quickly this loss of viral titre manifests itself, we chose an inactivation temperature of 60°C, which exhibited full inactivation after 15 min and representing a typical temperature to inactivate many different viruses, and measured inactivation over time. We observed an immediate significant loss of viral titres after 30 seconds (~700-fold) and near complete (~30,000-fold) inactivation after 1min (Fig 2D). At 5 or 15 min no infectious virus could be detected over the limit of detection (>125,000-fold).

Due to its proven thermolability, we next sought to determine for how long TULV particles retain their infectivity when stored at different temperatures. We stored virus aliquots at different temperatures (4°C, 21°C, 37°C) and in the absence of light and transferred viral samples after the indicated storage periods to -80°C and measured viral titres of all samples in parallel (Fig 2E). At 37°C, we observed an immediate significant loss of infectious viral titres after 24 h (~15-fold), which continued until infectious virus was last detected at 10 days (~6,000-fold reduction) and a complete loss of detectable infectious viral particles between 10 and 14 days, with an estimated half-life of approximately 6 h. At 21°C, we detected less than 20% reduction in viral titres after 24 h followed by a continued further loss resulting in an approximately ~20-fold reduction by day 42, with an estimated half-life of approximately 5 days. At 4°C, we barely detected any reduction of infectious viral titres after 9 weeks, resulting in an estimated half-life of approximately 531 days.

As viral particles rarely exist in solution for prolonged times in the environment, it is important to test the effect of dehydration on viral infectivity. We therefore air-dried TULV at room temperature on a sterile plastic surface inside a cell culture plate until virus suspensions have been fully dehydrated. At this point dehydrated virus samples were stored in the dark at either 4°C, room temperature (21°C) or 37°C, before viral particles were rehydrated at different time points using cell culture media and infectious virus titres were measured. The act of drying virus stocks itself resulted in significant loss of infectious virus titres with only 5–36% of viral particles remaining infectious immediately after dehydration (Fig 2F). When subsequently stored at 37°C, no infectious virus could be detected after 6 h. Storage at room temperature also resulted in a rapid loss of virus titres after 6 h (>100-fold), but low levels of infectious virus persisted and could be sporadically detected until 5 days, with no infectious virus detected after 7 days. Stored at 4°C, dehydrated virus stocks were able to maintain low levels of infectivity for 14 days, but underwent similar rapid reductions in infectious titres in the first days post-dehydration.

Overall, these data suggest, that while TULV can easily be inactivated at high (60°C) temperatures or by UV irradiation, it is able to retain significant infectivity at low temperatures or after freezing, potentially aiding its ability to survive outside of its host in the environment for long periods of time. In addition, even though dehydration of virus particles has a profound effect on viral infectivity, it could still be detected after several days at ambient temperatures and up to two weeks at low temperatures.

### Chemical inactivation of extracellular viral particles

We demonstrated above that TULV remains infectious on surfaces and suspended in liquids for considerable amounts of time and thus can pose a hazard to humans (Fig 2). In addition, many experimental techniques involve handling of liquids containing infectious virus. It is therefore important to gain a better understanding of how cell-free infectious material can effectively and safely be inactivated by chemical agents for further processing at lower biosafety conditions.

Therefore, we tested the ability of different chemical compounds commonly used in disinfectants and inactivation buffers to effectively reduce infectious viral titres in isolation. In brief, we incubated approximately 1x10^6^ plaque forming units (PFU) of TULV in a solution with the indicated chemical reagents for 10 min as detailed in Table 1. Subsequently we performed a buffer exchange of virus samples in order to reduce or eliminate potentially cytotoxic concentrations of chemical agents. Thusly purified virus solutions were titrated to determine infectious viral particle concentrations.

**Fig 3:**
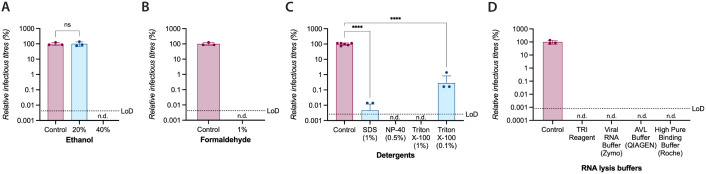
Chemical inactivation of cell-free TULV particles. Relative TULV titres after exposure to **(A)** ethanol, **(B)** formaldehyde, **(C)** detergents and **(D)** RNA lysis buffers at the indicated final concentrations for 10 min at room temperature. PBS was used as a non-inactivating control. All graphs represent relative data from immuno-plaque assays normalised to the control condition, with dots depicting three independent biological replicates (n = 3 for A, B and D; n = 6 for C) and error bars the standard deviation of the mean. The dotted lines represent the limit of detection (LoD) for each graph. Data points below the LoD are not shown and indicated as not detected (n.d.) in the absence of any measurable data points. Statistical significance was determined by one-way ANOVA followed by Dunnett’s correction for multiple comparisons (for A, C and D) or by an unpaired t-test (for **B)**: **** (p < 0.0001), *** (p < 0.001), ** (p < 0.01), * (p < 0.05), ns (p > 0.05).

We observed that incubation of virus in solution with ethanol at a final concentration of 40% resulted in complete (≥23,000-fold reduction) inactivation of virus infectivity, while 20% ethanol had no significant effect on viral titres (Fig 3A). Similarly, exposure of virus to formaldehyde at 1% final concentration also resulted in complete (≥23,000-fold reduction) inactivation of viral particles (Fig 3B).

Many experimental methods depend on the extraction of protein or nucleic acid contents for further analysis. We therefore tested the ability of typical lysis or extraction buffers for both protein- or RNA-based methods to fully inactivate TULV in a cell-free context. For protein extraction buffers we tested commonly used ionic (*e.g.,* SDS) and non-ionic (*e.g.,* NP-40 and Triton X-100) detergents that are commonly used to disrupt lipid cell membranes and lyse cells (Fig 3C). Out data showed that exposure to 0.5% NP-40 and 1% Triton X-100 fully inactivated (≥38,000-fold reduction in infectious viral titres) cell-free viral solutions, whereas exposure to 1% SDS (~21,000-fold reduction) or 0.1% Triton X-100 (~350-fold reduction) significantly, yet incompletely inactivated TULV within 15 min of exposure (Fig 3C). In addition, we tested commonly used commercial RNA extraction buffers, including TRI Reagent (Zymo Research), Viral RNA Buffer (Zymo Research), AVL Buffer (QIAGEN) and High Pure Binding Buffer (Roche) according to the manufacturers’ instructions (Fig 3D). Here, we could demonstrate full inactivation of viral particles (≥100,000-fold reduction in infectious viral titres).

These data demonstrate the how TULV particles can safely and effectively be inactivated in solution using commonly used chemical reagents.

### Chemical inactivation of infected cells

In Fig 3 we demonstrated that cell-free viral particles could efficiently be inactivated using commonly used buffers for cell fixation or RNA and protein extraction. Here, we tested the ability of different chemical compounds to inactivate TULV infected cells. In brief, we infected Vero E6 cells with TULV at an MOI of 0.001 for 9 days before removing cell culture supernatants and treating cells with the indicated chemicals (or PBS as a non-inactivating control) for 30 min at room temperature. After a buffer exchange to remove cytotoxic chemical concentrations, treated cells or cell lysates were subsequently transferred onto naïve Vero E6 cells and incubated for 7 days before measuring whether cells could be infected and infectious viral particles could be detected in cell culture supernatants, indicating that inactivation was incomplete. Since we demonstrated previously that the half-life of TULV at 37°C is extremely low, we are not comparing differences in measured viral titres but rather look for the any detection of virus as a measure for whether cellular inactivation was completely successful or not.

**Fig 4:**
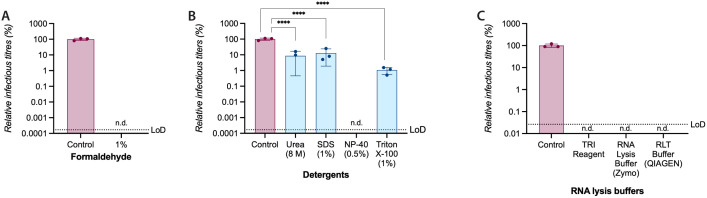
Chemical inactivation of TULV infected cells. Vero E6 cells were infected with TULV (MOI = 0.001) for 9 days. At 9 dpi, infected cells were inactivated using **(A)** formaldehyde, **(B)** detergents or **(C)** RNA lysis buffers at the indicated final concentrations for 30 min at room temperature. PBS was used as a non-inactivating control. Treated cells/lysates were used to infect naïve Vero E6 cells for 7 days. At 7 dpi, infectious viral titres were measured in the cell culture supernatant. All graphs represent relative data from immuno-plaque assays normalised to the control (PBS) condition, with dots depicting independent biological replicates (n = 3) and error bars the standard deviation of the mean. The dotted lines represent the limit of detection (LoD) for each graph. Data points below the LoD are not shown and indicated as not detected (n.d.) in the absence of any measurable data points. Statistical significance was determined by one-way ANOVA followed by Dunnett’s correction for multiple comparisons (for B and C) or by an unpaired t-test (for **A)**: **** (p < 0.0001), *** (p < 0.001), ** (p < 0.01), * (p < 0.05), ns (p > 0.05).

We found that fixation of cells with 1% formaldehyde fully inactivated TULV infected cells and prevented infection of naïve cells (Fig 4A). When using detergents commonly used in protein extraction buffers, we could not detect any infectious virus in the supernatant after inactivation with 0.5% NP-40, however, lysis of cells in 8 M urea, 1% SDS or 1% Triton X-100 was, albeit significantly reducing titres, not sufficient to fully inactivate TULV infected cells (Fig 4B). Cell lysis with commercial RNA extraction buffers, including TRI Reagent (Zymo Research), RNA Lysis Buffer (Zymo Research) and RLT Buffer (QIAGEN), led to a complete loss of detectable infectious viral titres (Fig 4C).

Together, these data complement our previous findings and establish which inactivation methods and buffers on their own are sufficient for intracellular inactivation of infectious material.

### Validation of inactivation methods using highly pathogenic hantaviruses

TULV is thought to be mostly apathogenic and therefore can safely be handled under biosafety level 2 conditions. Due to the genetic and structural similarities within the *Orthohantavirus* genus of the *Hantaviridae* family, it is a reasonable assumption that other members within this group – including highly pathogenic hantaviruses – will respond very similarly to these inactivation methods. We therefore tested whether Andes virus (ANDV), the prototypic HCPS-causing New World hantavirus, and Hantaan virus (HTNV), the prototypic HFRS-causing Old World hantavirus, displayed similar stability and could be efficiently and sufficiently inactivated by both high temperatures and chemical inactivation methods, which would allow inactivated samples to be transferred out of the biosafety level 3 facility.

**Fig 5:**
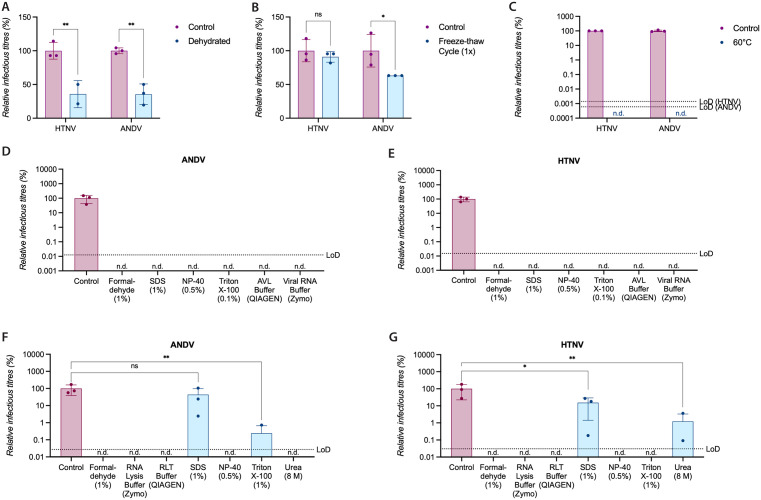
Validation of inactivation methods using highly pathogenic hantaviruses. **(A)** Relative infectious titres of HTNV or ANDV solutions before and after dehydration at room temperature. **(B)** Relative infectious titres of HTNV or ANDV before and after a single freeze-thaw cycle. **(C)** Relative infectious titres of HTNV or ANDV before and after incubation at 60°C for 5 min. **(D-E)** Relative infectious titres of **(D)** HTNV or **(E)** ANDV after exposure to the indicated chemicals at the indicated concentrations in solution for 10 min at room temperature. PBS was used as a non-inactivating control. **(F-G)** Vero E6 cells were infected with **(F)** ANDV or **(G)** HTNV (MOI = 0.001) for 12 days. At 12 dpi, infected cells were inactivated using the indicated reagents at the indicated final concentrations for 30 min at room temperature. PBS was used as a non-inactivating control. Treated cells/lysates were used to infect naïve Vero E6 cells for 9 days. At 9 dpi, infectious viral titres were measured in the cell culture supernatant. All graphs represent relative data from immuno-plaque assays normalised to the control condition, with dots depicting three independent biological replicates (n = 3) and error bars the standard deviation of the mean. The dotted lines represent the limit of detection (LoD) for each graph. Data points below the LoD are not shown and indicated as not detected (n.d.) in the absence of any measurable data points. Statistical significance was determined by two-way ANOVA followed by Šidák’s correction for multiple comparisons (for A-C) or by one-way ANOVA followed by Dunnett’s correction for multiple comparisons (for D-G): ** (p < 0.01), * (p < 0.05), ns (p > 0.05).

Initially, we tested whether both ANDV and HTNV exhibited similar stability under physical stresses as TULV. First, we dehydrated ANDV and HTNV at room temperature and measured infectious viral titres before and after dehydration (Fig 5A). We observed that for both viruses a similar proportion of infectious viral particles remained immediately after dehydration (~35%) as for TULV (Fig 2F). Next, we compared the effects of a single freeze-thaw cycle on virus stocks (Fig 5B). Here, we observed for HTNV a non-significant reduction in infectious titres, whereas ANDV titres dropped significantly (~37%), similarly to TULV (Fig 2B). Finally, we also validated that exposure to 60°C temperatures for 5 min was sufficient to fully inactivate both ANDV and HTNV similar to TULV (Fig 5C).

Next, we performed chemical inactivation of cell-free ANDV (Fig 5D) and HTNV (Fig 5E) as previously shown for TULV. Analogous to TULV, we also observed a complete loss of infectious viral titres using 1% formaldehyde, 0.5% NP-40, AVL Buffer and Viral RNA Buffer for both HTNV (≥6,500-fold reduction) and ANDV (≥8,000-fold reduction). Notably, we also observed full inactivation using 1% SDS, 0.1% Triton X-100 and 8 M urea treatment, which in contrast only resulted in significant yet incomplete inactivation using TULV (Fig 3C).

Finally, we validated whether complete inactivation of ANDV or HTNV infected Vero E6 cells could be achieved using fixation or commonly used cell lysis buffers. Here, we infected Vero E6 cells at an MOI of 0.001 for 12 days, treated cells for 30 min at room temperature with the indicated chemicals and transferred cells or cell lysates onto naïve cells. After 9 days we measured whether we could detect any infectious viral particles in the cell culture media (Fig 5F-5G). Here, we demonstrated that exposure to 1% formaldehyde, RNA Lysis Buffer, RLT Buffer and 0.5% NP-40 resulted in complete inactivation of cells infected with both viruses. Inactivation with 1% SDS was not sufficient for intracellular inactivation of either virus. Inactivation with 1% Triton X-100 (ANDV) and 8 M urea (HTNV) also resulted in incomplete inactivation for one virus each.

Overall, we were able to demonstrate that highly pathogenic hantaviruses such as ANDV and HTNV exhibit similar stability as TULV when exposed to environmental stresses or inactivating chemicals. We evaluated the ability of several buffers commonly used for different downstream analysis methods to inactivate highly pathogenic hantaviruses and identified safe, effective and reliably inactivating methods that could be used for different analysis methods (Table 3).

**Table 3:** Efficacy of tested virucidal methods.

	*Inactivation method*	*Final concentration*	*Exposure time*	*Virus*	*Efficacy* *(log*_*10*_ reduction) *	*Inactivation*
Suspension inactivation	Heat (42°C)	N/A	15 min	TULV	0.1	Incomplete
	Heat (50°C)	N/A	15 min	TULV	0.4	Incomplete
	Heat (56°C)	N/A	15 min	TULV	5.0	Incomplete
	Heat (60°C)	N/A	15 min	TULV	≥5.0	Complete
	Heat (60°C)	N/A	5 min	TULV, ANDV, HTNV	≥5.0, ≥ 3.9, ≥ 3.8	Complete
	UV-C (254 nm)	300 mJ/ cm^2^	N/A	TULV	0.8	Incomplete
		1,500 mJ/ cm^2^	N/A	TULV	1.7	Incomplete
		4,500 mJ/ cm^2^	N/A	TULV	2.2	Incomplete
	Ethanol	20%	10 min	TULV	0	Incomplete
		40%	10 min	TULV	≥4.4	Complete
	Formaldehyde	1%	10 min	TULV, ANDV, HTNV	≥4.4, ≥ 3.9, ≥ 3.8	Complete
	Sodium dodecyl sulfate (SDS)	1%	10 min	TULV	4.3	Incomplete
	Sodium dodecyl sulfate (SDS)	1%	10 min	ANDV, HTNV	≥3.9, ≥ 3.8	Complete
	Nonidet P-40 substitute (NP-40)	0.5%	10 min	TULV, ANDV, HTNV	≥4.6, ≥ 3.9, ≥ 3.8	Complete
	Triton X-100	0.1%	10 min	TULV	2.5	Incomplete
	Triton X-100	0.1%	10 min	ANDV, HTNV	≥3.9, ≥ 3.8	Complete
	Triton X-100	1%	10 min	TULV	≥4.6	Complete
	TRI Reagent	3:1 (75%)	10 min	TULV	≥4.1	Complete
	Viral RNA Buffer (Zymo Research)	2:1 (66.67%)	10 min	TULV, ANDV, HTNV	≥4.6, ≥ 3.9, ≥ 3.8	Complete
	Buffer AVL (QIAGEN)	4:1 (80%)	10 min	TULV, ANDV, HTNV	≥4.6, ≥ 3.9, ≥ 3.8	Complete
	Binding Buffer (Roche)	2:1 (66.67%)	10 min	TULV	≥4.6	Complete
Cellular inactivation	Formaldehyde	1%	30 min	TULV, ANDV, HTNV	≥5.8, ≥ 3.6, ≥ 3.5 *	Complete
	Urea	8 M	30 min	TULV, HTNV	1.1, 1.9 *	Incomplete
	Urea	8 M	30 min	ANDV	≥3.6 *	Complete
	Sodium dodecyl sulfate (SDS)	1%	30 min	TULV, ANDV, HTNV	0.9, 0.4, 0.8 *	Incomplete
	Nonidet P-40 substitute (NP-40)	0.5%	30 min	TULV, ANDV, HTNV	≥5.8, ≥ 3.6, ≥ 3.5 *	Complete
	Triton X-100	1%	30 min	TULV, ANDV	2.0, 2.6 *	Incomplete
	Triton X-100	1%	30 min	HTNV	≥3.5 *	Complete
	TRI Reagent	100%	30 min	TULV	≥3.6 *	Complete
	RNA Lysis Buffer (Zymo Research)	100%	30 min	TULV, ANDV, HTNV	≥3.6, ≥ 3.6, ≥ 3.5 *	Complete
	Buffer RLT (QIAGEN)	100%	30 min	TULV, ANDV, HTNV	≥3.6, ≥ 3.6, ≥ 3.5 *	Complete

* For cellular inactivation assays, efficacy values are not representative of the actual degree of cellular inactivation, but rather a binary indicator for whether inactivation was complete or incomplete. The actual virus titres and reduction rates are highly dependent on the surviving amount of infectious material and the time point of sampling.

## Discussion

Emerging viruses have caused several global pandemics and local epidemic outbreaks in the past decades. Hantaviruses represent a particular threat to global health, due to their high prevalence in their natural host species, the ubiquitous abundance of their rodent host species and their close interactions with human populations, the highly pathogenic and lethal disease in humans as well as the proven potential for human-to-human transmission. Our need to study these viruses to understand basic molecular details as well as to develop effective and safe antivirals and vaccines is thus self-evident.

Hantaviruses are primarily transmitted environmentally: viral particles are shed in bodily fluids and secretions such as rodent droppings, which might subsequently be accidentally ingested or inhaled by humans or other host species. Hantaviruses therefore rely on a certain virion stability to withstand environmental stresses in a cell-free context, such as dehydration or freezing of viral particles or prolonged exposure to high or low temperatures.

We demonstrated that when stored in cell culture media, TULV remains remarkably stable after 9 weeks of storage at 4°C with an estimated half-life of 531 days. When stored at room temperature, low levels of infectious virus could still be detected after 6 weeks (with an estimated half-life of 5.3 days), whereas at 37°C it rapidly loses its infectious potential with a half-life of 6 h, but can still be detected at low levels for up to 10 days (Fig 2E). These observations explain why the cumulative infectious viral titres in cell culture when growing TULV is not significantly different from the titres that were only produced in the last day or days (Fig 1B). These data are also somewhat consistent with a previous study, where it was demonstrated that HTNV can remain infectious for up to 7 days in cell culture media when stored at 37°C or for up to 9 days when stored at 21°C, but for up to 12 weeks when stored at 4°C [15]. Another study showed that PUUV and TULV lost their infectivity after 24 h when stored at 37°C, after 11 days when stored at 23°C and still retained reduced infectivity after 18 days when stored at 4°C [14]. It is important to note though, that the previously published results for PUUV and TULV only were able to capture a very small dynamic range of virus titres (less than 50-fold reduction of infectivity), compared to several orders of magnitude (approximately 100,000-fold reduction of infectivity) that can be detected using immuno-plaque assays as used in the previously published HTNV study and in our own data. This might explain why infectious viral particles could be detected for longer periods of time by us using the same virus (TULV) compared to the previously published results. Interestingly, it was also demonstrated that PUUV remains infectious to naïve bank voles in bank vole droppings for up to 15 days [14]. While not equivalent to being fully solubilised in cell culture media, it is likely that the virus is at least partly protected from full dehydration and other stresses inside animal droppings. Another interesting aspect of these findings is that hantaviruses are unlikely to survive *in vivo* outside of infected cells for long periods of time as the elevated temperature alone would result in a rapid loss of infectivity.

As viral particles seldom remain suspended in liquid for prolonged times in the environment, we showed that dehydration of TULV, HTNV and ANDV particles results in a similar (~65%) loss of infectivity (Figs 2F and 5A). These results broadly concur with previous studies, where on the one hand, an approximately 10-fold reduction in infectious viral particles were observed after a few hours and no infectious virus remaining after 24 h for HTNV, PUUV and TULV [14,15]. And on the other hand, we also recently demonstrated that while ANDV particles undergo an approximately 1,000-fold reduction in infectivity after storage at room temperature for 24 h when dehydrated on steel discs, it can nevertheless remain infectious for more than 5 days, similar to our data for TULV which we could detect at low concentrations for up to 5 days at room temperature and up to 14 days at 4°C [18]. At 37°, however, dehydrated TULV did not retain any infectivity after 6 h. Slight differences between the studies could perhaps be explain by different experimental setups, different methods for virus preparation and other environmental factors that are known to influence viral stability and that were not taken into account in these studies, such as humidity, light exposure, pH level and protein content of the virus stocks [33–37].

Many hantaviruses can only be handled in a high containment facility and need to be fully inactivated before they can be processed under normal biosafety levels. While inactivation methods have been extensively tested against a multitude of different viruses [38,39], there are not many studies which comprehensively and convincingly validate the safe inactivation of cell-free and cell-associated hantaviruses. This is of particular importance, since state-of-the-art experimental procedures require highly divergent inactivation and lysis buffers and previous studies have mainly focused on disinfectants and fixatives. We demonstrate that hantaviruses rapidly lose their infectivity after heat inactivation at 60°C and that after 30 seconds at 60°C infectious titres already are approximately 1,000-fold reduced and that no infectious particles remain after 5 min (Figs 2C-2D and 5C). Exposure to 56°C resulted in significant yet incomplete inactivation of TULV (Fig 2C). A previous study showed that in solution PUUV is fully inactivated after 15 min at 56°C [14]. This discrepancy can be explained by the significantly higher limit of detection in that study which would not allow the detection of low levels of infectious virus such as in our study. Interestingly, the same study also suggests that PUUV is able to withstand 56°C heat for up to 2 h when fully dehydrated, which appears to be in direct contradiction with our own data demonstrating that TULV rapidly loses its infectivity even at 37°C (Fig 2F).

Recently, we already established that ANDV can effectively and quickly be inactivated using ethanol or 2-propanol-based WHO-recommended hand rub formulations [18], which was in line with previous studies using enveloped RNA and DNA viruses [33,40–42]. Furthermore, PUUV has been shown to be effectively inactivated using commercial disinfectants Clidox, Halamid-d, and Virkon S [16]. In addition, cell-free HTNV was shown to be fully inactivated by 2 min exposure to 40% ethanol [15] and intracellular HTNV was fully inactivated using 100% methanol (8 min), 1% paraformaldehyde (20 min) or 1:1 acetone/methanol (10 min) [13]. Another study also demonstrated inactivation of PUUV with exposure to 0.025% formaldehyde [17]. Our own results confirm that full inactivation of hantaviruses can be achieved using 40% ethanol (cell-free virus) or 1% formaldehyde (cell-free and cell-associated virus) (Figs 3A-3B, 4A, 5D-5G). In addition, we also provide evidence for the complete inactivation of hantaviruses using different commercial RNA extraction kits (Figs 3D, 4C, 5D-5G) and different detergents – including SDS, NP-40, Triton X-100 and urea - used in common lysis buffers (Figs 3C, 4B, 5D-5G). Interestingly, our results suggest that ANDV and HTNV are slightly more susceptible to SDS and Triton X-100 in solution than TULV, which exhibited partial resistance to complete inactivation using 1% SDS or 0.1% Triton X-100 alone. While more studies would be needed to investigate these differences, it is also important to highlight that we only tested single reagents in isolation and that commonly combinations of different inactivation methods are used, *e.g.,* several inactivating reagents in lysis buffers or a combination of lysis buffers and heat inactivation. For intracellular inactivation of infectious material, we noticed that SDS (1%), Triton X-100 (1%) or urea (8 M) treatment alone was not sufficient for full inactivation of hantaviruses, while RNA extraction buffers and NP-40 (0.5%) were sufficient (Figs 4B and 5F-5G). This is an important finding, demonstrating what lysis buffer components represent safe inactivation methods for highly pathogenic hantaviruses if a combination with heat inactivation is impossible for downstream procedures. This also highlights the need to carefully evaluate each new inactivation method and to perform heat inactivation in combination with chemical inactivation where possible.

Overall, these data are broadly in line with similar studies validating inactivation methods for other highly pathogenic RNA viruses, including arenaviruses and Nipah virus [38,43,44]. However, our study often establishes a lower necessary baseline for inactivation compared to many traditional inactivation methods, indicating that highly pathogenic viruses often are inactivated at much lower concentrations or exposure times than commonly used in the laboratory.

While laboratory-acquired human cases of hantavirus infections are very rare, there have been documented cases involving both laboratory animals and pets [45,46]. It is therefore important to develop safe and effective disinfection and inactivation protocols for laboratory procedures. Furthermore, to study highly pathogenic viruses and perform different downstream analyses outside of biosafety level 3 or 4 facilities, it is essential to establish inactivation protocols that are compatible with these analyses. Our data establish safe and effective inactivation methods compatible with many common downstream molecular biology, cell biology, immunology or virology assays, including nucleic acid extraction, western blots, co-immunoprecipitation assays, ELISA, immunofluorescence assays and flow cytometry. Crucially, we demonstrate that our findings are not only applicable to a single hantavirus species, but we that are broadly valid across a diverse set of Old World and New World hantaviruses, including the highly pathogenic HTNV and ANDV. Moreover, our data also goes a long way to detail the environmental stability of hantavirus particles with strong implications for our understanding of the environmental transmission potential of hantaviruses.

## Supporting information

S1 Table Data tables for figures. Data tables with absolute or normalised virus titres that each graph is based on.
(XLSX)
